# Ethnomedicinal Knowledge of Traditional Healers in Roi Et, Thailand

**DOI:** 10.3390/plants9091177

**Published:** 2020-09-10

**Authors:** Auemporn Junsongduang, Wanpen Kasemwan, Sukanya Lumjoomjung, Wichuda Sabprachai, Wattana Tanming, Henrik Balslev

**Affiliations:** 1 Department of Science and Technology, Faculty of Liberal Arts and Science, Roi Et Rajabhat University, Thailand, Selaphum, Roi Et 45120, Thailand; kasemwan1591@gmail.com (W.K.); sukayyalacumcang425@gmail.com (S.L.); Thungko.bio@gmail.com (W.S.); 2 Queen Sirikit Botanical Garden, Mae Rim, Chiang Mai 50180, Thailand; w.tanming@gmail.com; 3 Ecoinformatics and Biodiversity, Department of Biology, Aarhus University Build 1540, Ny Munkegade 114, 8000 Aarhus C, Denmark; henrik.balslev@bios.au.dk

**Keywords:** herbalist, traditional knowledge, ethnobotany, northeastern Thailand

## Abstract

Traditional healers in Thailand are a primary source of health care for the Thai people. Highly experienced traditional healers are generally older people and they continue to pass away without recording or passing on their knowledge. Consequently, the cumulative knowledge held by traditional healers regarding the use of medicinal plants is being eroded and could be lost. In this study, we aimed to identify and document the medicinal plants and associated ethnobotanical knowledge held by traditional healers in Roi Et in northeastern Thailand. Data and plant specimens were collected from four traditional healers of the Phu Tai people. They were selected by purposive sampling and questioned using a semi-structured interview. The interviews covered their training, the ailments treated, treatment techniques, method of preparation and in addition, several healing sessions were observed. During field walks, we searched for the medicinal plants with the healers to review and document the availability of medicinal plants at each locality and in different habitats around the villages. Use values (UV) were calculated to estimate the importance of each medicinal plant and informant agreement ratios (IAR) were calculated to understand how widely known the uses were. The four Phu Tai traditional healers knew 162 medicinal plant species in 141 genera and 63 families. The family with the most medicinal plants was Leguminosae with 15 species. The plant part that they used most commonly was the stem, which was used for 82 species (49%). The most common preparation method was decoction, which was done for 124 species (75%). The most important and widely used medicinal plants were *Rothmannia wittii*, which had the highest use value (UV = 1.7). Most medicinal plants were used for treating tonic (34 species (21%)). Jaundice had the highest informant agreement ratio (IAR = 0.5). The most common life form among the medicinal plants was trees (56 species (34%)). The medicinal plants were mostly collected in community forests (81 species (49%)). Considering the richness of the healer’s pharmacopeia, and the fact that their profession is not being perpetuated, this study points to the urgent need to document the traditional knowledge from the old herbalists before it disappears with the last practitioners from rural communities in Thailand.

## 1. Introduction

Many countries have their own traditional systems of healing that usually depend on local folk remedies and traditional medicine that meets their needs to treat ailments [1,2,3,4]. Traditional medicine is also part of the rapidly growing alternative health systems in westernized societies, and its economic importance is becoming increasingly recognized. However, traditional medicine is of enormous importance in developing countries, where it is usually firmly embedded in broader belief systems [5]. The 2014–2023 Strategy for Traditional Medicine (TM) from the WHO posits that traditional treatments, traditional practitioners, and herbal medicines are the main source of health care, and often the only one, for millions of people [6].

In many parts of the world, such as in Iraq [1], Italy [7], Ghana [8], northwestern Algeria [9], and Ethiopia [10], expenditure on traditional medicine is not only significant, but growing rapidly. The traditional medicinal knowledge and practices in many developing countries have not been adequately studied, exploited, or documented [11]. These traditional knowledge systems, transmitted orally from one generation to the next among traditional health practitioners, are in danger of disappearing due to poor relations between older and younger generations [12,13,14]. Assessing these products and ensuring their safety and efficacy through registration and regulation are major challenges. In Asia, people still use traditional medicine as a result of historical circumstances and cultural beliefs [5]. In China, traditional medicine accounts for some 40% of all health care delivered [5]. It has been estimated that about 35,000 to 70,000 plant species are used for medicinal purposes globally, of which 6500 species are utilized in Asia [15]. Recognition by governments of their clinical and pharmaceutical value is growing, although this varies widely between countries [16].

Traditional medicine is one of the local wisdoms in rural Thai communities. This knowledge has been accumulative through experiences by the process of learning, selecting, improving, developing, and perpetuating [17]. This knowledge has been used as a self-reliant system for preventing Thai people from sickness, for taking care of themselves, and for adapting to the environment and the changing times [17]. Astonishingly, 2187 plant species were reported as medicinal in Thailand in a recent study [18]. The advancement of medicine in Thailand during the first half of the 20th century led to the decline of the practice of traditional medicine. Nevertheless, the paradigm for Thai national health policy, as put forward in the Tenth National Health Development Plan (2007–2011), shifted to a community-based approach, focusing on providing care that is responsive to peoples’ needs. In the plan, local wisdom and existing health resources are recognized as important resources in Thailand’s healthcare. This was incorporated in the National Health Act as a guideline for health policymaking and practice [19]. The idea of using local wisdom for healthcare also reflects the link between people’s activity and their environment. It implies that people can create their own healthcare network by relying on local resources and sharing their knowledge and experience to better care for themselves, their families and other community members.

In local communities, traditional healers are part of culture and tradition, and continue to have a high social standing in most communities, exerting influence on local health practices. It is, therefore, worthwhile exploring the possibilities of engaging them in primary health care and training them accordingly [20]. Traditional healers in Thailand, called Mor Baan, were the primary source of health care for Thai people before the introduction of western medicine [21].

Recently, modern health professionals have held a key role as health care providers in the Thai health care system, but traditional healers and their practice have not disappeared from Thai society, especially in remote parts of the country [22]. Traditional healers in Thailand have been formally accepted as primary healthcare (PHC) providers since the late nineteenth century. Today, they still have an active role in the health of the Thai population [23]. Traditional healers can be found in all parts of Thailand [24,25]. A survey in 2006 was conducted across 75 provinces and found a total of 27,760 traditional healers in Thailand [21]. The northeastern region had the highest number, with 14,146 practitioners. These healers serve customers at home, in places of business, or at work, and some also work in local government health centers [25]. According to the policies of the Thai government, stated by the parliament on 23 March 2005, Thai traditional healers have become a part of national health policy. The Thai government tries to develop, transfer, and protect the wisdom of Thai traditional medicine, indigenous medicine, alternative medicine, and medicinal plants through primary healthcare by Tambon health promoting hospitals [26].

A crucial issue concerns the transfer of knowledge relating to Thai traditional medicine, which is continually declining [27]. Highly experienced traditional healers are generally older people and they continue to pass away, usually without recording or passing on their knowledge. Secondly, many of the younger generations of medical practitioners have low regard for traditional medicine and are drawn to other occupations because of job security and higher salaries. Finally, the forests of Thailand are being decimated, which suggests that the medicinal plants necessary for traditional healers are becoming less available. Consequently, the knowledge of the standard healers regarding the utilization of medicinal plants is being diminished and will possibly be lost before being explored through systematic studies [27]. For traditional healers, there are high hopes that the information gathered by researchers from the local universities, on the plants and traditional methods they use, will result in affirmation and recognition of their practices [28].

The primary objective of this study was to identify and document the medicinal plants and associated ethnobotanical knowledge of traditional healers. It is hoped that the plants they use will be further studied in the future, especially their phytochemistry and pharmacology. The present study focuses on documenting the diagnosis of diseases in general, details of the utilization of medicinal plants, and the criteria for selecting medicinal plants. The ethnomedicinal information presented here was collected from four highly experienced traditional healers and we hope that it will benefit people who are interested in traditional medicine and many associated aspects of medicinal plants. In this context and departing from the Roi Et experience, we aim at answering the following specific questions: (1) What is the traditional knowledge used by Phu Tai healers for treating their patients? (2) How many medicinal plants do they know and use for treating patients in their community? (3) Which species are most used? (4) How many ailments are treated with traditional medicinal plants? (5) Which plant parts are commonly used for medicine? (6) How many preparation methods are available? (7) What is the similarity of medicinal plant species used among traditional healers? (8) What are the habits and habitats of medicinal plants?

## 2. Results

### 2.1. The Traditional Healers

The four healers together knew 162 species of medicinal plants belonging to 141 genera in 63 families. The average number of medicinal species known by the healers was 68 species (Table 1).

### 2.2. Diversity of Medicinal Plants

The family with most medicinal species was Leguminosae, which had 15 spp. of medicinal plants (9.2%) followed by Rubiaceae (11 spp., 6%), Zingiberaceae (9 spp., 5.5%), and Euphobiaceae (8 spp., 5%). Apocynaceae, Acanthaceae and Rutaceae had seven (4.3%), six (3.7%), and five (3%) spp. respectively. Annonaceae, Bignonaceae, Ebenaceae, Malvaceae and Phyllanthaceae all had four spp. (2.4%) (Table S1). The remaining families only had three (1.8%) or fewer species.

### 2.3. Similarities of the Medicinal Plants Used by Healers

Comparison of the four healers with Jaccard’s similarity index showed that they used very different sets of plants for treating people. Only 4–8% of the used plants were shared by any pair of healers (Table 2.)

### 2.4. Life Forms of the Plants Used

Five life forms of medicinal plants were used by the four healers, the most common one being trees that were represented by 56 spp. (34%) followed by shrubs 47 spp. (29%), climbers 38 spp. (23%), and herbs 21 spp. (12%).

### 2.5. Plant Parts Used

The most commonly used plant part was the stem, which was used for almost half of the species (82 spp. (49%)), followed by the root (59 spp. (36%)), the leaf (31 spp. (19%)), bark (20 spp. (12%)), fruit (14 spp. (8%)), rhizome (8 spp. (5%)), flower and shoot (4 spp. each (2% each)), while whole plant, sap and seed were used in only 2 spp. each (1% each).

### 2.6. Preparation of Medicine

The healers described 12 different methods that they used to prepare plant medicines. The most common preparation method was decoction, followed by crushing or grinding, and applying the medicine to the skin. Grinding the plant with water and drinking the solution and soaking the plant to produce a bath were other common methods of preparing medicine among the healers. The least common methods of preparation were to grind the plant with water and then use the medicine to wash the hair, to grind the medicinal plant with lemon juice and drink it, and to chew the plant. These rare methods were used for a single plant, which is less than one percent of the used species (Table 3).

### 2.7. Use Value Index (UV) and Symptoms and Ailments Treated by the Phu Tai Traditional Healers

The most important and widely used medicinal plants species were *Rothmannia wittii* (Craib.) Bremek. (Rubiaceae) with a use value (UV) of 1.75 followed by *Tinospora crispa* (L.) Hook. f. and Thomson (Menispermaceae) with a use value (UV) of 1.5, while *Cissampelos pareira* L. (Menispermaceae), *Gardenia saxatilis* Geddes. (Rubiaceae), *Ixora lucida* R.Br. ex Hook. f. (Rubiaceae), *Memecylon edule* Roxb. (Memecylaceae), *Ochna integerrima* (Lour.) Merr. (Ochnaceae), and *Bombax anceps* Pierre. (Bombacaceae) all had use values (UV) of 1. The total number of ailments and symptoms treated by the four traditional healers was 51. Most medicinal plants were used for treating tonic (35 spp., 21%), followed by fever (31 spp., 19%) and diarrhea (20 spp., 12%) (Table S1).

### 2.8. Informant Agreement Ratio (IAR) among Healers

The use category with the most use-reports was that of plants used to treat jaundice with an IAR value of 0.5 (seven use-reports, four spp.), which showed the highest degree of consensus. This was followed by the detoxicant category with an IAR value of 0.37 (nine use-reports, six spp.), the insect bite/head lice category with an ICF value of 0.33 (four use-reports, three spp.), the gastritis category with an IAR value of 0.30 (14 use-reports, 10 spp.) and the tonic category with an IAR value of 0.26 (39 use-reports, 29 spp.).

### 2.9. Habitats and Status of Medicinal Plants

The healers collected most of their medicinal plants from the community forests surrounding their villages (81 spp., 50%) followed by forest protected by the government *Phu Dan Suang*, (56 spp., 34%), home gardens (38 spp., 23%), and around the village (8 spp., 5%). Swamps were the least used habitat for collecting medicinal plants with only one species (<1%). The majority of medicinal plants were native to Thailand, while, 18 spp. of medicinal plants were exotic (11%). Most of the exotic plants were found in their home garden (10 spp., 5%), followed by community forest (5 spp., 2%) (Table S1).

## 3. Discussions

### 3.1. The Traditional Healers

Generally, traditional healing is a gender-based practice, although in some communities both men and women are practitioners [29]. In Thailand, most traditional healers are men [30], which was also the case in this study. Thai culture assumes that the traditional role of the man in a family is as a leader and primary breadwinner, so men are expected to provide for their families, while the woman care for the children. Traditional healers are very important in Thai society, so men always aspire for that position. Most people known to have substantial knowledge and experience with practicing traditional medicine are old [31]. Normally, the number of medicinal plants known by a healer increases with age and decreases with increasing number of years of formal schooling [32]. What we found here is different from that general rule. The number of medicinal plant species known was not related to the age of the informant and older herbalists knew fewer medicinal plants than the younger ones. It could be that some herbalists treat only certain conditions such as cancer or postpartum conditions in women. Furthermore, some of them had experienced loss of memory because of their age, which suggests that the process of declining biocultural knowledge is a complex matter and is not only driven by young peoples’ lack of interest in learning from the older generation. As for the learning of medicinal knowledge, some healers reported that they learned about medicinal plants following their parents and grandparents when they gathered remedies in the forest when they were young, which was similar to what was found in a recent study of the Mien in northern Thailand [32]. The traditional knowledge of the interviewed healers had been transferred from their parents, but they also had other sources of knowledge such as friends or formal training. Apart from knowledge about plants, the traditional healers also knew about minerals and animals (snail, horn, bone) that formed part of the treatments as previously reported among other cultures [33]. Furthermore, in this study traditional healers also practiced incantations and ceremonies as part of the treatments.

### 3.2. Diversity of Medicinal Plants

Together the healers knew 162 species of medicinal plants, the most popular ones belonging to Leguminosae, Rubiaceae, and Zingiberaceae. Another study of the same ethnic group, but in Nakhonphanum province, reported 176 plants that were used for medicinal purposes [34], 53 of which were the same as in our study. In Nakhonphanum province, the most commonly used plant family was again Leguminosae, which was followed by Euphorbiaceae. Elsewhere in Thailand and among other ethnic groups, Leguminosae is the plant family with the most species used as medicine and with the most records of use [27,28,29,30,31,32,33,34,35]. Other families found to be important in this study, such as Rubiaceae, Zingiberaceae and Euphorbiaceae, are, like the legumes, large tropical families [36], and they are common and species-rich in Thailand. Species of Leguminosae are commonly used as medicinal plants for treating fever, tonic, and diabetes [36,37]. For example, *Caesalpinia sappan* L. is used in Thailand and elsewhere to treat a large number of medicinal conditions. It is used in Kerala as a part of a herbal drink to quench thirst, as a blood purifier, an antidiabetic, and to improve complexion and several other conditions [38]. Another important medicinal plant family is Zingiberaceae, which is widely used in traditional medicine in many Asian countries such as Laos, Cambodia and Thailand [39]. In Thailand and Laos, species of Zingiberaceae (mainly species of *Kaempferia* and *Zingiber*) have strongly aromatic rhizomes and are commonly used medicinal plants [40]. In northeastern Thailand, especially in Roi Et, traditional healers have cultivated or collected these plants in their home garden. Species of Zingiberaceae are considered to be very powerful medicinal plants. Their medicinal functions have been broadly discussed and accepted, and are used in many traditional recipes [41] including, for instance, species such as *Alpinia zerumbet* (Pers.) B.L. Burtt and R.M. Sm. and *Alpinia galanga* (L.) Willd. In this survey, the traditional healers used Zingiberaceae rhizomes for digestive disorders. Finally, Rubiaceae is used as an anti-inflammatory [42]. Several pharmacological studies have confirmed that plants from these families are efficient in the treatment of a variety of health conditions and symptoms [43,44,45,46,47].

### 3.3. Similarities of Plants Used by the Healers

The values of Jaccard’s similarity index for medicinal plants used by the healers suggest that they possessed different traditional knowledge relative to diagnosis, herbal prescription, and making suggestions in health care, although they lived in nearby areas. Even for the same species of medicinal plant, the four healers sometimes used them for different purposes. For example, *Flacaurtia indica*, which was used to treat gastritis by one healer and to promote lactation in women during the postpartum period by another healer (Table S1). Their different knowledge was based on experience received discretely from their families and adapted by themselves. Such differences in medicinal plant knowledge on a very local scale have also been documented in Songkhla province in southern Thailand [36] and just north of our study area in Kalasin province [30]. Thus, it is very important to collect a variety of knowledge from the local healers before it disappears from their community.

### 3.4. Life Forms of Medicinal Plants

Though not often reported on, a preponderance of trees and shrubs over other life forms such as climbers and herbs is commonly found in ethnobotanical studies [31,48]. The proportion of species in each life form may simply be a reflection of their proportions of the general flora, although we do not have the life form statistics for the areas around the study villages or for Thailand as a whole. One study of traditional uses of legumes among the Karen in Thailand, however, showed that the proportion of each life form of used legumes was similar to the proportion of the same life forms for legumes in general in the Thai flora [49].

### 3.5. Plant Parts Used for Medicine

Many ethnomedicinal studies include information about the plant parts used for medicine. In a meta-analysis of 64 ethnomedicinal studies from all over Thailand, the most commonly used parts were stems and leaves followed by roots, and entire plants [18], but there was some variation, such as in Nakhon Phanom where leaves were the most commonly used part [34]. The use of different plant parts should be taken into account when estimating the effects of medicinal plants on plant populations, especially because the use depends on differences in quantities of phytochemical compounds in different parts of a plant [50]. In some cases, dried wood or roots can maintain their bioactive compounds for a longer time after harvesting than leaves can [18]. Moreover, environmental conditions may affect the traditional uses of plants. In wetter areas, plants produce more leaves, and therefore harvesting leaves could be less detrimental to the plant. In contrast, it may be more difficult to get enough leaves to make medicine in the dryer areas, so bark, wood, and roots would be preferred. Some medical conditions can be treated by whole plants because several parts of the plant contain similar active compounds and pharmaceuticals or have similar bioactive properties that are useful in the treatment of a particular symptom [50]. For instance, as documented in this study, gastritis and flatulence are treated with whole plants of the small sized bamboo *Vietnamosasa pusilla* (A.Chev. and A.Camus) T.Q.Nguyen (Table S1). However, the efficacy of medicinal plants depends on the content of secondary metabolites in each species, and also by the part used, the season and the period of collecting.

### 3.6. Preparation and Administration

The medicinal plants were prepared and administered using a variety of methods. The most common preparation method used was decoction in water followed by oral ingestion. In this respect, our results agree with those obtained in many other ethnomedicinal studies [18,35,51]. In general, water is the most commonly used solvent for extracting medicinal compounds from plants [51,52]. Other methods using different solvents included alcohol infusion, fixed oil extraction, and mixing with lime juice [18]. The frequent administration method of oral ingestion is most likely because it is an easy way to administer the medicine [53], and because it allows the practitioner to adjust the taste of the medicine. For example, in the case of very bitter medicine, the healer may add sugar or honey to make the ingestion more pleasant [18]. Oral ingestion is the most common way of using medicine in both northern [51,52,53,54] and southern Thailand [27]. Decocting and boiling the plant is used elsewhere to prepare herbal medicines for postpartum women [51]. The second most common method for preparing the plant medicines was to crush or grind the plant and then apply it to the skin or wound. Similar to our observations, the crushing or grinding method is commonly used elsewhere when the medicine must be applied directly to an affected organ [55]. A particular method of preparation that we observed was to grind stems or bark with water or lime juice and drink the solvent.

Bathing with boiled water containing the plant extract was used by the healers in this study to treat asthmatic fevers and swelling, and elsewhere it was used to drive away fatigue [56]. Bathing has a refreshing effect caused by the smell of volatile oils from the plants [18]. Based on the healer’s prescription, they usually boiled the herbs until the extract attained a dark color. Once the water containing the medicinal extract was warm, they would use it for a bath once or twice a day. The residue was boiled with water again for a second time and used repeatedly until its color and odor had diminished [51]. In northern Thailand, medicinal plants are sometimes used directly as part of daily life activities such as cooking food or eating fruits or snacks [51,57], but since this study was focused on healers, such daily use of medicinal plants was not systematically observed. However, the constraints of medicinal plants, such as mistaken medicinal plant species and mistaken preparation, must be taken into consideration. Adverse reactions from the misuse of medicinal plants are partly due to misunderstandings about the species, because some species of medicinal plants are similar or have the same common name, and some are poisonous. This is why traditional healers are so important for the rural community. If there is a lack of expertise in classifying medicinal plants, it can lead to bodily harm from their misuse.

### 3.7. Use Value Index (UV) and Symptoms and Ailments Treated

The most important and most common medicinal plant species used by the healers was *Rothmannia wittii* Craib (Bremek.) (Rubiaceae) as demonstrated by its high use value index of 1.75. The healers used this species in a decoction to treat failing lactation in postpartum women, as a tonic, and to treat muscle pain. This species is a widely used Thai medicinal plant called “Muk Mor” in Thai language. It is otherwise used for treatment of venereal disease and to treat fever [58], and as an anti-inflammatory agent [59]. The second most used plant was *Tinospora crispa* (L.) Hook. f. and Thomson (Menispermaceae; UV = 1.5), which is also used in other parts of Thailand and in the Philippines as a stem extract to treat jaundice, cholera, malaria, fevers, stomach trouble, indigestion, diarrhea, and infections by worms in children [35,36]. Furthermore, traditional healers in Malaysia, Guyana, Bangladesh, and India use this plant to treat diabetes [60]. In a study of the same ethnic group as in this study in Nakhon Phanom, the most used medicinal plant was *Crinum asiaticum* L. var. *asiaticum* (Amaryllidaceae). There, it was used to treat injuries [34] but this species was not even recorded in our study, which demonstrates how the use of medicinal plants is often very local and the property of only a few healers. The difference in traditional knowledge in each region possibly depends on their history and the time their community was settled, or by the experiences and activities in their daily lives [51,61].

The medicinal plants recorded here were used to treat 52 different ailments. Most plants were used to treat tonic, followed by fever and swellings. This ailment was also the most common in nearby Nakhon Phanom [34]. Here we use the word “tonic” to mean the continuous contraction of muscles. Herbal medicines that are used for tonic may be defined as plants ingested to extend wellness, and help restore tone and invigorate systems within the body, or to promote general health and well-being. Fever is one of the oldest known clinical indicators of disease within the mammalian host, one of the most common reasons for medical consultations worldwide and quite common in rural communities. Fever often occurs as a response to infection, inflammation, or trauma. Apart from being a regulated rise in body temperature, fever is often accompanied by other symptoms such as changes in metabolic and physiological characteristics of body systems and alterations in immune responses. The pathogenesis of fever and febrile response depends on the clinical presentation and outcome of the varied illnesses and diseases [62]. It occurs when the organs, skin, or other parts of the body enlarge, and occur internally. It can be affect the outer skin and muscles. A range of conditions can cause swelling, such as insect bites, illnesses, or injuries. These often occur during the villagers’ daily lives or working on the farm.

### 3.8. Informant Agreement Ratio (IAR)

The category with most use-reports was that of plants used to treat jaundice, which also had the highest degree of consensus among the healers. Otherwise healthy newborns in Thailand have a high risk of having jaundice [63]. In India, 82 species have medicinal folklore reports, are commonly used in traditional systems as antimicrobials and are supposedly effective in the treatment of jaundice. In Thailand, some Thai medicinal plants, such as the liquorice *Glycyrrhiza glabra* (L.) (Leguminosae) can reduce bilirubin, which is the cause of jaundice symptoms by glycyrrhizin and glycyrrhizic acid [64]. Here, we found that healers also used some medicinal plants to treat jaundice including *Tinospora crispa* (L.) Hook. f. and Thomson (Menispermaceae), *Xantonnea parviflora* (Kuntze) Craib. (Rubiaceae), and *Morinda coreia* Buch.-Ham. (Rubiaceae). However, the high IAR values suggest a well-defined selection criterion in the community and/or information exchange between informants [65].

### 3.9. Habitats and Status of Medicinal Plants

The majority of medicinal plants known by the healers were collected from community forests. Some of them were cultivated as a medicinal plant in the home-gardens or on their farm when the natural habitat was located far away from their village. In addition, the majority of exotic medicinal plants in this study were found in the home-gardens. The use of exotic plants may be because they are widespread and easy to access in their community [66]. However, in this study, the healers always cultivated medicinal plants because they could not be found in the forest, such as *Agave sisalana* Perrine (Asparagaceae) and *Ananas bracteatus* (Lindl.) Schult. and Schult. f., and some were used as food, such as *Allium sativum* L. and *Piper nigrum* L. Some studies have shown that habitats are the most important for the collection of medicinal plants. For instance, in nearby Nakhon Phanom, cultivated fields were more important than natural forests [34]. However, the report from Phattalung province in southern Thailand found that most medicinal plants were gathered from the forest [27], as we found in our study. Today, medicinal plants are also threatened by habitat destruction and the change from subsistence farming to cash crops. Especially in northeastern Thailand, cash cropping was widely introduced to the agricultural system, cultivating such species as rubber (*Hevea brasiliensis* Müll.Arg., Euphorbiaceae), cassava (*Manihot esculenta* Crantz, Euphorbiaceae), and sugarcane (*Saccharum officinarum* L., Poaceae). Therefore, the medicinal plants and the traditional knowledge of their uses are currently endangered.

## 4. Materials and Methods

### 4.1. Study Area

The northeastern region, also known as Isan, located on the Khorat Plateau, is the largest in Thailand, limited by the Mekong River along the border with Laos, and divided into 20 provinces. Northeastern Thailand covers 170,000 km^2^ and has more land dedicated to agriculture than the rest of the country (9.25 million hectares) [67,68]. Approximately 17 million people, corresponding to 30% of the Thai population live in the region [69] and 94% of them live in rural areas [67]. Isan is the poorest region of Thailand. The Isan people are very religious and influenced by nearby Cambodia and Laos, and, relative to the rest of Thailand, Isan culture and food is unique. The Isan people are ethnically of Tai-Lao origin, constituting one of the largest minorities in the country, followed by the Phu Tai ethnic group. The main languages spoken are Thai, Lao, and ethnic languages. Most northeastern Thai speak a dialect of Lao mixed with some influences from Thai, also known as Isan [70]. Agriculture, the largest sector of the economy, accounts for 22% of gross regional product, compared to 8.5% for Thailand as a whole. Rice is the main crop occupying 60% of the cultivated land. However, farmers are increasingly diversifying into cash crops such as sugarcane and cassava, which are cultivated on vast scales, and to a lesser extent, rubber [67]. However, agricultural production per area remains low, due to the relatively dry climate and the often saline soils.

**Figure 1:**
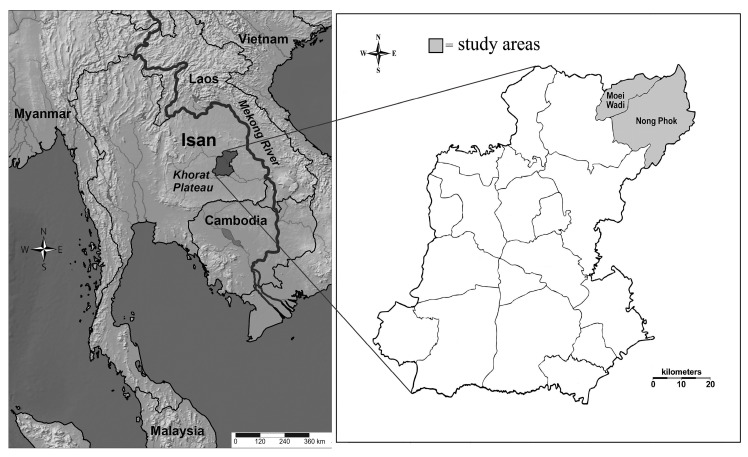
Location of Roi Et province in Thailand where ethnobotanical data concerning medicinal plants were collected in Moei Wadi and Nong Phok districts.

This study was undertaken in the Roi Et province (8299 km^2^) in northeastern Thailand (Figure 1) where the traditional practices of healers still exist and remain extremely important. We documented traditional knowledge on medicinal plants in traditional practices from four traditional healers of the Phu Tai ethnic group. Phu Tai is a small tribe of Tai people of which there are many in Asia including Taidam, Taidang, Taikhao, Tailao, Taipuan, Taiyai, Tainung, and Taimoei [71].

### 4.2. Informants

We found four old traditional Phu Tai healers whose practices were still alive (Table 1). They were all male and 76–82 years old; two in Moei Wadi districts (16°03′ N 103°39′ E) and two in Nong Phok district (16°03′ N 103°39′ E) (Figure 1). All four healers were Buddhist and they had only attended primary school. Two of them had been trained by family members, and the other two had received training in herbal medicine by other healers. Three of the healers had more than 60 years of experience in treating people (Table 1). All of them were farmers and practiced healing secondary to their main occupation.

### 4.3. Data Collection

Data and plant specimens were collected from April 2015–April 2016. The traditional healers were selected by purposive sampling [72]. The healers were visited in their homes and informal meetings were held in Isan or Phu Tai languages. Semi-structured interviews were performed to collect qualitative and quantitative data on the medicinal plants. All four healers were interviewed about their knowledge, training, ailments, treatment techniques, and method of preparation, and the interviews were supplemented by direct observation. Field walks searching for the medicinal plants were made to review and document the availability of medicinal plants in different habitats in and around the villages. We recorded information about local names of the plants, plant parts used for treatments, method of preparation, route of administration, and the habitats of medicinal plants. Voucher specimens were collected to document botanical identification. Preliminary identifications of medicinal species using vernacular names were made by the healers in the field. Species were initially identified on the basis of their common names, following Tem Smitinand′s Thai Plant Names [73]. Subsequently, some of the voucher specimens were brought to the herbarium of the Queen Sirikit Botanical Garden (QBG) for taxonomic confirmation based on the Flora of Thailand. Comparison with existing collections was performed by W.T. The voucher specimens were collected in forested areas adjacent to the villages and in home gardens. The vouchers are kept in the Department of Science and Technology, Roi Et Rajabhat University, Roi Et province, Thailand.

### 4.4. Data Analysis

Here, we define “use-report” as the mention of a particular plant being used for one particular purpose by one particular informant in a particular place at a given time. The use value is a quantitative measure that demonstrates the relative importance of the species. The use value index (*UV*) [74] is defined as:

*UV* = *Ui*/*N* (1)

where *Ui* is the number of use-reports cited by each informant for a given species and *N* is the total number of informants. *UVs* are low (approaching 0) when there are few use-reports related to the use of a species. *UVs* are high (approaching 1) when there are many use-reports for a species, implying that the plant is commonly known by the informants.

The Informant Agreement Ratio is an index for testing homogeneity of knowledge [75]

*IAR* = *Nur* − *Nt*/(*Nur* − 1) (2)

where *Nur* refers to the number of use-reports for a particular use category and *Nt* refers to the number of taxa used for a particular use category by all informants. *IAR* values are low (approaching 0) if plants are used randomly or if there is no exchange of information about their use among informants. *IAR* values are high (approaching 1) when there is a well-defined selection criterion in the community and/or if information is exchanged between informants [75].

We used Jaccard’s Index (*JI*) to determine the similarity of medicinal plants species used by the four healers [76]. This index is based on the presence or absence of species on each list. Relating the number of shared species to the total number, the index is expressed as:

(3) JI=c⁄(a+b+c) × 100

where *a* is the number of species unique to healer *a*, *b* is the number of species unique to healer *b*, and *c* is the number of species used by both healers.

## 5. Conclusions

Over the last decade, the official attitude in Thailand towards traditional medicine has changed, and the policy is now to integrate traditional medicine into the public health system. However, many doctors trained in western medicine in Thailand remain skeptical towards traditional medicine, although the Thai public is showing an increasing interest in its healing powers. The present study shows that traditional healers possess rich ethnopharmacological knowledge. They have important local knowledge of many species used to treat a variety of ailments. There is an urgent need to document this valuable knowledge of medicinal plants in northeastern Thailand. Raising awareness, more education on protection, conservation and sustainable use of medicinal plant resources are all needed for training the villagers. Ethnobotanical studies should be considered as a way to collect and support communities in their efforts to maintain and conserve their traditional knowledge. As a result, this study helps to identify many highly valuable medicinal plant species with high potential for economic development through the sustainable collection. The medicinal plants found to have high use values should be subjected to pharmacological studies to validate their use and to isolate their bioactive compounds for further study. Antibacterial and antioxidant activities will be examined in some medicinal plants identified from this research as the next step.

## Figures and Tables

**Table 1:** Profile of the four Phu Tai healers interviewed in Roi Et province.

District	Name (Age)	Years Practicing Herbal Medicine	Number of Known Medicinal Plant (spp.)	Type of Training
Nong Phok	Healer 1 (78 years)	64	89	Family members
	Healer 2 (82 years)	60	37	Formal Training
Muei Wadi	Healer 3 (76 years)	45	95	Formal Training
	Healer 4 (77 years)	60	53	Family members
Average	78	57	68	

**Table 2:** Jaccard’s similarity index showing the overlap of the medicinal plants known by the four healers.

Healer	Healer 2 Species (%)	Healer 3 Species (%)	Healer 4 Species (%)
Healer 1	11 (6.5)	14 (8.0)	11 (8.5)
Healer 2	-	9 (7.8)	3 (4.4)
Healer 3	-	-	8 (7.9)

**Table 3:** Preparation methods for medicinal plants used by four healers in Roi Et, Thailand.

Methods	Number of Species	%
Decoction	124	76
Crush or grind and apply to skin	23	14
Grind with water and drink	10	6
Soaked and bath	6	3
Eat as food	4	2
Soaked and drink	4	2
Eat as fresh	3	1
Boil and bath	2	1
Steamed	2	1
Chewed	1	<1
Grind with water and wash hair	1	<1
Grind with lemon juice and drink	1	<1

## Supplementary Materials

The following are available online at https://www.mdpi.com/2223-7747/9/9/1177/s1, Table S1: Medicinal plants used by four Phu Tai healers in Roi Et province, Thailand.
