# Supplementary material for: Ethnomedicinal Knowledge of Traditional Healers in Roi Et, Thailand
Source: Plants (Basel). 2020 Sep 10;9(9):1177. doi: 10.3390/plants9091177 (PMC7570034; doi:10.3390/plants9091177)
Supplement: Supplementary file 1 [file plants-09-01177-s001.pdf]

Table S1. Medicinal plants used by four Phu Tai healers in Roi Et province, Thailand.

No.	Species (Auemporn Junsongduang Vourcher no.)	Family	Local name	Symptoms and Ailments treated (Part used)	Preparation	Life forms and status	Source of Medicinal plant	UV
1	*Acalypha indica* L. (AJ-58)	Euphorbiaceae	Tam yae maeo	Tuberculosis (L), Asthma (R)	Decoction	H	Community forest	0.50
2	*Acanthus ebracteatus* Vahl (AJ-59)	Acanthaceae	Ngueak pla mo	Diabetes (S)	Decoction	Shr	Protected forest	0.25
3	*Acronychia pedunculata* (L.) Miq. (AJ-105)	Rutaceae	Ka uam	Headache (S)	Decoction	Shr	Community forest	0.50
4	*Aganonerion polymorphum* Spire (AJ-187)	Apocynaceae	Som lom	Fever (R)	Decoction	C	Home garden	0.25
5	*Agave sisalana* Perrine (AJ-60)	Asparagaceae	Pan son na rai	Swelling (R)	Decoction	ExShr	Home garden	0.25
6	*Allium sativum* L. (AJ-106)	Amaryllidaceae	Kra thiam	Hemorrhoids (L)	Mashed and applied to skin	ExH	Home garden	0.25
7	*Alpinia galanga* (L.) Willd. (AJ-188)	Zingiberaceae	Kha	Flatulence (R)	Eaten as food	H	Community forest	0.25
8	*Alpinia zerumbet* (Pers.) B.L.Burtt & R.M.Sm. (AJ-61)	Zingiberaceae	Kha Khom	Muscle pain (Sh), Flatulence (Sh)	Decoction	H	Community forest, Protected forest	0.75
9	*Amphineurion marginatum* (Roxb.) D.J.Middleton (AJ-107)	Apocynaceae	Khruea sai tan	Urination difficulty (S)	Decoction	C	Community forest	0.25
10	*Anacardium occidentale* L. (AJ-186)	Anacardiaceae	Ma muang him ma phan	Diarrhea (S)	Decoction	ExT	Home garden	0.25
11	*Ananas bracteatus* (Lindl.) Schult. & Schult. f. (AJ-189)	Bromeliaceae	Mak nut	Leucorrhea (R), Urination difficulty (R)	Decoction	ExH	Home garden	0.50

No.	Species (Auemporn Junsongduang Vourcher no.)	Family	Local name	Symptoms and Ailments treated (Part used)	Preparation	Life forms and status	Source of Medicinal plant	UV
12	*Annona squamosa* L. (AJ-185)	Annonaceae	Buk khiap	Abscess (F)	Mashed apply to skin	ExShr	Community forest	0.50
13	*Aphanamixis polystachya* (Wall.) R. Parker (AJ-190)	Meliaceae	Toom tang	Body pain (S)	Decoction	T	Community forest, Protected forest	0.25
14	*Aporosa villosa* (Wall. ex Lindl.) Baill. (AJ-184)	Euphorbiaceae	Mueat yai	Diarrhea (S), Fever (S)	Decoction	T	Community forest	0.50
15	*Aquilaria crassna* Pierre ex Lecomte (AJ-62)	Thymelaeaceae	Krit sa na	Tonic (L,S)	Decoction	T	Community forest	0.25
16	*Artocarpus lacucha* Buch.-Ham. (AJ-191)	Moraceae	Ma hat	Aphthous ulcer (S)	Chewed	T	Community forest	0.50
17	*Asparagus racemosus* Willd. (AJ-214)	Asparagaceae	Chi kohk	Swelling (R)	Decoction	C	Community forest	0.25
18	*Baccaurea parviflora* (Müll. Arg.) Müll. Arg. (AJ-108)	Phyllanthaceae	Ma fai pa	Convulsion (R)	Grind with water and drink	T	Community forest	0.25
19	*Barleria lupulina* Lindl. (AJ-104)	Acanthaceae	Salet phang phon	Hemorrhoid (S) Insect bite (S) , Herpes zoster (L), Tonic (S)	Decoction, Mashed apply to skin	ExShr	Community forest	1.00
20	*Barringtonia racemosa* (L.) Spreng. (AJ-183)	Lecythidaceae	Chik	Leucorrhea (B,S)	Decoction	Shr	Community forest	0.25
21	*Bauhinia malabarica* Roxb. (AJ-215)	Fabaceae	Som siao	Gastritis (L,S)	Decoction	T	Home garden	0.25
22	*Bauhinia strychnifolia* Craib (AJ-216)	Fabaceae	Ya nang daeng	Detoxification (L), Tonic (R)	Decoction	WC	Protected forest	0.75
23	*Bombax anceps* Pierre (AJ-64)	Malvaceae	Ngio pa	Tonic (S), Snake bite (B, R), Swelling (L)	Decoction	T	Protected forest	1.00
**No.**	**Species (Auemporn Junsongduang Vourcher no.)**	**Family**	**Local name**	**Symptoms and Ailments treated (Part used)**	**Preparation**	**Life forms and status**	**Source of Medicinal plant**	**UV**

24	*Caesalpinia godefroyana* Kuntze (AJ-213)	Fabaceae	Nam han	Diarrhea (S)	Decoction	C	Community forest	0.25
25	*Caesalpinia sappan* L. (AJ-103)	Fabaceae	Fang	Tonic (S,F), Asthma (B,S)	Decoction	T	Around village	0.50
26	*Calamus acanthophyllus* Becc. (AJ-193)	Arecaceae	Wai nang	Tonic (Rh)	Decoction	WC	Protected forest	0.25
27	*Canarium subulatum* Guillaumin (AJ-194)	Burseraceae	Ma kok	Mouth ulcers (B,F,S) , Fever (R)	Decoction	T	Protected forest	0.75
28	*Capparis micracantha* DC. (AJ-63)	Capparaceae	Chai chu	Erectile dysfunction (S)	Decoction	Shr	Protected forest	0.25
29	*Capparis sepiaria* L. (AJ-109)	Capparaceae	Nam wua sang	Asthma (R)	Grind with water and drink, Boiled and bath	WC	Community forest	0.25
30	*Casearia grewiifolia* Vent. (AJ-180)	Salicaceae	Pha sam	Jaundice (S)	Decoction	T	Around village	0.25
31	*Cassia fistula* L. (AJ-110)	Fabaceae	Khun	Postpartum women disorders (B,S)	Decoction	T	Home garden	0.25
32	*Catunaregam tomentosa* (Blume ex DC.) Triveng. (AJ-195)	Rubiaceae	Nam thaeng	Leucorrhea (S) , Diarrhea (S)	Decoction	Shr	Community forest, Protected forest	0.50
33	*Celastrus paniculatus* Willd. (AJ-65)	Celastraceae	Khruea mak taek	Leucorrhea (S)	Decoction	WC	Protected forest	0.25
34	*Cereus hexagonus* (L.) Mill. (AJ-112)	Cactaceae	Ta bong phet, Dab ngeuak	Gastritis (S)	Steam and Decoction	ExShr	Protected forest	0.25
35	*Chromolaena odorata* (L.) R.M.King & H.Rob. (AJ-196)	Asteraceae	Sap suea	Wound (L), Diabetes (R), Diarrhea (R)	Mashed apply to skin, Decoction	ExH	Home garden, Around village	0.75

No.	Species (Auemporn Junsongduang Vourcher no.)	Family	Local name	Symptoms and Ailments treated (Part used)	Preparation	Life forms and status	Source of Medicinal plant	UV
36	*Cinnamomum iners* Reinw. ex Blume (AJ-179)	Lauraceae	Op choei	Tonic (B,S)	Eat as food	T	Community forest, Protected forest	0.25
37	*Cissampelos pareira* L. (AJ-111)	Menispermaceae	Ma noy	Tonic (L), Scurvy (L), Morning sickness (L)	Eat as fresh	C	Community forest	1.00
38	*Clausena harmandiana* (Pierre) Guillaumin (AJ-66)	Rutaceae	Song fa	Spider bite (L), Cough (R), Sore throat (R)	Mashed apply to skin, Decoction	Shr	Community forest	0.75
39	*Clausena wallichii* Oliv. (AJ-102)	Rutaceae	Song fa	Swelling (R)	Soaked for Bath and drink	Shr	Protected forest	0.25
40	*Clerodendrum schmidtii* C. B. Clarke (AJ-113)	Lamiaceae	Puang pee kao	Fever (R)	Grind with water and drink	Shr	Home Garden	0.25
41	*Clitoria ternatea* L. (AJ-101)	Fabaceae	Anchan	Dandruff (Fl), Eye vision (R)	Mashed apply to skin, Decoction	ExC	Home Garden	0.50
42	*Coccinia grandis* (L.) Voigt (AJ-197)	Cucurbitaceae	Tam lueng	Body odor (F)	Mashed apply to skin,	H	Community forest	0.25
43	*Connarus semidecandrus* Jack (AJ-212)	Connaraceae	Keng Khruea/ Khruea ma wo	Muscle pain (S)	Decoction	WC	Home Garden	0.25
44	*Cheilocostus speciosus* (J.Koenig) C. D. Specht (AJ-67)	Costaceae	Ueang mai na	Swelling (R)	Soaked and Bath	C	Protected forest	0.25
45	*Cratoxylum cochinchinense* (Lour.) Blume (AJ-100)	Hypericaceae	Tio kliang	Wound (S)	Mashed apply to skin, Decoction	T	Community forest	0.25

No.	Species (Auemporn Junsongduang Vourcher no.)	Family	Local name	Symptoms and Ailments treated (Part used)	Preparation	Life forms and status	Source of Medicinal plant	UV
46	*Cratoxylum formosum* (Jacq.) Benth. & Hook. f. ex Dyer *(AJ-211)*	Hypericaceae	Tio dang	Paralysis (R), Leucorrhea (R)	Decoction	T	Community forest	0.50
47	*Croton crassifolius* Geiseler (AJ-178)	Euphorbiaceae	Phang khi	Flatulence (L,R)	Decoction	Shr	Community forest	0.50
48	*Croton persimilis* Müll. Arg. (AJ-217)	Euphorbiaceae	Plao yai	Gastritis (L,S)	Decoction	Shr	Home Garden	0.25
49	*Curcuma aromatica* Salisb. (AJ-198)	Zingiberaceae	Pha ya wan	Dysentery (S)	Decoction	ExH	Home Garden	0.25
50	*Curcuma* sp. (AJ-114)	Zingiberaceae	Ka min kao	Leucorrhea (Rh)	Decoction	H	Home Garden	0.25
51	*Curcuma zanthorrhiza* Roxb. (AJ-199)	Zingiberaceae	Wan chak motluk	Postpartum women disorders (Rh)	Decoction	ExH	Home Garden	0.25
52	*Datura metel* L. (AJ-99)	Solanaceae	Ma khuea ba	Abscess (R), Sedative (R)	Grind with water and drink	ExShr	Protected forest	0.50
53	*Dialium cochinchinense* Pierre (AJ-218)	Fabaceae	Keng	Fever (S)	Decoction	T	Community forest	0.25
54	*Dillenia hookeri* Pierre (AJ-210)	Dilleniaceae	San tia	Fever (R)	Decoction	Shr	Community forest	0.25
55	*Dioecrescis erythroclada* (Kurz) Tirveng. (AJ-219)	Rubiaceae	Mui dang	Leucorrhea (S), Diarrhea (S)	Decoction	T	Protected forest	0.50
56	*Diospyros ehretioides* Wall. ex G. Don (AJ-115)	Ebenaceae	Tong toong/Tab tao	Postpartum women disorders (S)	Decoction	T	Community forest, Protected forest	0.50
57	*Diospyros filipendula* Pierre ex Lecomte (AJ-68)	Ebenaceae	Kan chong	Asthma (R), Hemorrhoids (S)	Grind with water and drink, Decoction	T	Community forest	0.50
58	*Diospyros mollis* Griff. (AJ-177)	Ebenaceae	Mak kluea	Worm infection (F), Laxative (F)	Decoction	T	Community forest	0.25

No.	Species (Auemporn Junsongduang Vourcher no.)	Family	Local name	Symptoms and Ailments treated (Part used)	Preparation	Life forms and status	Source of Medicinal plant	UV
59	*Diospyros rhodocalyx* Kurz (AJ-116)	Ebenaceae	Paya chang dam	Swelling (B, S)	Soaked for drink and Bath	ST,T	Protected forest	0.25
60	*Dipterocarpus obtusifolius* Teijsm. ex Miq. (AJ-98)	Dipterocarpaceae	Heang	Diarrhea (S, B)	Decoction	T	Community forest	0.25
61	*Droogmansia godefroyana* (Kuntze) Schindl. (AJ-117)	Fabaceae	Tong mong	Wound (L)	Mashed and apply	Shr	Community forest	0.25
62	*Elaeagnus latifolia* L. (AJ-136)	Elaeagnaceae	Khruea mak lod	Muscle pain (S)	Decoction	C	Protected forest	0.25
63	*Elephantopus scaber* L. (AJ-69)	Asteraceae	Fai nok khum	Tonic (R), Impotence (L)	Decoction	H	Protected forest	0.25
64	*Ellipanthus tomentosus* Kurz (AJ-157)	Connaraceae	Ta nok kot	Tonic (S)	Decoction	ST,T	Protected forest	0.25
65	*Enkleia malaccensis* Griff. (AJ-137)	Thymelaeaceae	Po tao hai	Diarrhea (B,R)	Decoction	WC	Community forest, Protected forest	0.25
66	*Erythrophleum succirubrum* Gagnep. (AJ-138)	Fabaceae	Phan sat	Fever (B, S)	Decoction	T	Protected forest	0.25
67	*Erythroxylum cuneatum* (Miq.) Kurz (AJ-158)	Erythroxylaceae	Hun hai	Wound (Sh)	Mashed apply to skin, Decoction	T	Around village	0.50
68	*Euphorbia hirta* L. (AJ-70)	Euphorbiaceae	Nam nom ratchasi	Urination difficulty (S), Lactation (R)	Decoction	H	Protected forest	0.50
69	*Eurycoma harmandiana* Pierre (AJ-200)	Simaroubaceae	Pla lai phueak	Aids (R)	Decoction	Shr	Community forest, Protected forest	0.25
70	*Eurycoma longifolia* Jack (AJ-71)	Simaroubaceae	Pla lai phueak	Abscess (R), Leucorrhea (S,R)	Decoction	Shr	Community forest, Home garden	0.5

No.	Species (Auemporn Junsongduang Vourcher no.)	Family	Local name	Symptoms and Ailments treated (Part used)	Preparation	Life forms and status	Source of Medicinal plant	UV
71	*Ficus foveolata* Buch.-Ham. ex Sm. (AJ-176)	Moraceae	Ma gra teup rohng	Tonic (S)	Decoction	WC	Community forest, Home garden	0.25
72	*Flacourtia indica* (Burm.f.) Merr. (AJ-201)	Salicaceae	Mak ban	Gastritis (F,S,L) Lactation (S)	Decoction	T	Community forest	0.50
73	*Getonia floribunda* Roxb. (AJ-97)	Combretaceae	Tin tung krue	Fever (S), Asthma (B)	Decoction	WC	Protected forest Community forest	0.25
74	*Gardenia saxatilis* Geddes (AJ-118)	Rubiaceae	Khoi dan	Fever (R), Tonic (R), Intoxication (R, S, L)	Grind with water and drink	Shr	Protected forest Community forest	1.00
75	*Glycosmis pentaphylla* (Retz.) DC. (AJ-135)	Rutaceae	Chom chuen	Cancer (S), Postpartum women disorders (S), Headache (L)	Decoction	Shr	Protected forest Community forest	0.75
76	*Goniothalamus laoticus* (Finet & Gagnep.) Bân (AJ-140)	Annonaceae	Khao lam	Muscle pain (S)	Decoction	Thr	Community forest, Home garden	0.25
77	*Harrisonia perforata* (Blanco) Merr. (AJ-134)	Simaroubaceae	Nam go ta	Muscle pain (S), Fever (B)	Decoction	Shr	Community forest, Home garden	0.50
78	*Hedyotis capitellata* Wall. ex G. Don (AJ-72)	Rubiaceae	Ya mung kratai	Swelling (S, L)	Soaked and Bath, Soaked and Drink	WC	Around village, community forest	0.25
79	*Helicteres angustifolia* L. (AJ-119)	Malvaceae	Pow ke kai	Diabetes (R)	Decoction	Shr	Community forest	0.25
80	*Heliotropium indicum* L. (AJ-120)	Boraginaceae	Ya kaung chang	Gallstones (S,L,F) Cough (S), Mouth ulcer(L,S)	Decoction	H	Protected forest Community forest	0.75

No.	Species (Auemporn Junsongduang Vourcher no.)	Family	Local name	Symptoms and Ailments treated (Part used)	Preparation	Life forms and status	Source of Medicinal plant	UV
81	*Hoya kerrii* Craib(AJ-141)	Apocynaceae	Buap lom, Hua jai tod sa gan	Cancer (S, L)	Decoction	WC	Community forest	0.25
82	*Irvingia malayana* Oliv. ex A.W.Benn. (AJ-159)	Irvingiaceae	Ga bok wan	Tonic (S)	Decoction	T	Community forest	0.25
83	*Ixora lobbii Loudon* (AJ-202)	Rubiaceae	Kem dang	Urination difficulty (R)	Decoction	Shr	Community forest	0.25
84	*Ixora lucida* R.Br. ex Hook.f. (AJ-175)	Rubiaceae	Kem kao	Fever(R), Lactation problem (L, S)<br>Blood building (F, R)	Decoction	Shr	Community forest	1.00
85	*Jatropha curcas* L. (AJ-96)	Euphorbiaceae	Luk dum	Fever (S)	Decoction	ExShr, ST	Around village, Community forest	0.25
86	*Jatropha gossypifolia* L. (AJ-95)	Euphorbiaceae	Sa bo dang/Buk yao	Wound (Sa)	Mashed and apply to skin	ExShr	Protected forest Community forest	0.25
87	*Justicia adhatoda* L. (AJ-142)	Acanthaceae	Hu ha	Fever (L)	Mashed and apply to skin	Shr	Home garden	0.25
88	*Kaempferia galanga* L. (AJ-156)	Zingiberaceae	Naeng hom	Gastritis (Rh)	Decoction	H	Home garden	0.25
89	*Kaempferia roscoeana* Wall. (AJ-203)	Zingiberaceae	Pan din yen	Swelling (R), Gastritis (R)	Decoction	H	Community forest	0.50
90	*Lagerstroemia calyculata* Kurz (AJ-209)	Lythraceae	Pleuay	Fever (B)	Decoction	T	Protected forest	0.25
91	*Lagerstroemia duperreana* Pierre ex Gagnep. (AJ-160)	Lythraceae	Puei	Flatulence (B)	Decoction	T	Protected forest, Community forest	0.25
92	*Leea indica* (Burm. f.) Merr. (AJ-73)	Vitaceae	Tang gai dang	Abscess (R)	Grind and apply to pok	ST/Shr	Protected forest, Community forest	0.25
93	*Leucaena leucocephala* (Lam.) de Wit (AJ-74)	Fabaceae	Ka tin	Fever (S)	Decoction	Shr/ST	Community forest	0.25

No.	Species (Auemporn Junsongduang Vourcher no.)	Family	Local name	Symptoms and Ailments treated (Part used)	Preparation	Life forms and status	Source of Medicinal plant	UV
94	*Litsea cubeba* (Lour.) Pers. (AJ-121)	Lauraceae	King kai ton	Tonic (B,S)	Decoction	T	Community forest	0.25
95	*Litsea glutinosa* (Lour.) C.B.Rob. (AJ-143)	Lauraceae	Mai mee	Abscess (B,L,R)	Mashed and apply to pok	T	Home Garden	0.50
96	*Luffa cylindrica* (L.) M.Roem. (AJ-122)	Cucurbitaceae	Buab	Dandruff (F)	Mashed apply to head	H	Community forest	0.25
97	*Lygodium flexuosum* (L.) Sw. (AJ-162)	Lygodiaceae	Moy sao gae	Gallstones (R)	Decoction	WC	Home Garden	0.25
98	*Mansoa alliacea* (Lam.) A. Gentry (AJ-144)	Bignoniaceae	Gra tiam tao	Intoxication (R), Fever (R)	Grind with Lemon juice and drink	ExC	Protected forest, Community forest	0.50
99	*Memecylon edule* Roxb. (AJ-94)	Melastomataceae	Muad ae	Tonic (S), Lactation (S), Cancer (S,L), Fever (R)	Decoction	Shr	Protected forest, Community forest	1.00
100	*Microcos tomentosa* Sm. (AJ-155)	Malvaceae	Kao je	Allergy (R)	Decoction	T	Protected forest	0.25
101	*Micromelum minutum* Wight & Arn. (AJ-174)	Rutaceae	Samud, Hud sa kun	Spider bites (R)	Grind and apply to wound	Shr	Around village	0.25
102	*Millingtonia hortensis* L.f. (AJ-75)	Bignoniaceae	Dok peep	Tuberculosis (S), Hemorrhoids (nose) (Fl,R)	Decoction	T	Home garden	0.50
103	*Momordica charantia* L. (AJ-76)	Cucurbitaceae	Mara kee nok	Swelling (F), Fever (F)	Mashed and apply to skin,Decoction	H	Protected forest	0.50
104	*Morinda coreia* Buch.-Ham. (AJ-123)	Rubiaceae	Yaw pa	Diabetes (S,R), Jaundice (B,S)	Decoction	T	Protected forest, Community forest	0.50

No.	Species (Auemporn Junsongduang Vourcher no.)	Family	Local name	Symptoms and Ailments treated (Part used)	Preparation	Life forms and status	Source of Medicinal plant	UV
105	*Morinda citrifolia* L. (AJ-93)	Rubiaceae	Yaw pa	Abscess (S), Tonic (S), Postpartum women disorder (S)	Mashed and apply to skin, Decoction	T	Protected forest	0.75
106	*Myxopyrum smilacifolium* (Wall.) Blume (AJ-163)	Oleaceae	Nao duan ha	Fever (S)	Decoction	WC	Swamp	0.25
107	*Nelumbo nucifera* Gaertn. (AJ-92)	Nelumbonaceae	Bue luang	Nasal polyps (Fl)	Mashed and apply to skin	AqH	Protected forest, Community forest	0.25
108	*Ochna integerrima* (Lour.) Merr. (AJ-145)	Ochnaceae	Paya chang san, Chang nao	Lactation (S), Tonic (S), Fever (S)	Decoction	T	Home garden	1.00
109	*Oroxylum indicum* (L.) Kurz (AJ-133)	Bignoniaceae	Ma lin mai	Diarrhea (B,S,F,Fl,)	Decoction	T	Community forest	0.25
110	*Oxyceros horridus* Lour. (AJ-164)	Rubiaceae	Kad kao	Muscle pain (S)	Decoction	WC	Community forest	0.25
111	*Parinari anamensis* Hance (AJ-124)	Chrysobalanaceae	Pok	Swelling (S)	Soaked and Bath, Soaked and Drink	T	Community forest	0.50
112	*Passiflora foetida* L. (AJ-204)	Passifloraceae	Kreua etok	Diarrhea (F)	Eat as fresh	ExC	Around village	0.25
113	*Peltophorum dasyrrhachis* (Miq.) Kurz. (AJ-220)	Fabaceae	A rang	Gallstones (S), Muscle pain(S)	Decoction	T	Around village	0.50
114	*Phoenix acaulis* Roxb. (AJ-221)	Arecaceae	Pang	Swelling (Rh)	Decoction	T	Community forest	0.25
115	*Phyllanthus elegans* Wall. ex Müll.Arg. (AJ-173)	Phyllanthaceae	Puk wan dong	Body pain (S)	Steamed	H	Community forest	0.25
116	*Phyllanthus reticulatus* Poir. (AJ-154)	Phyllanthaceae	Gang pla	Fever (S)	Soaked and Bath	Shr	Protected forest	0.25
117	*Phyllodium elegans* (Lour.) Desv. (AJ-208)	Fabaceae	Gret lin noi	Fever (S), Teeth (R)	Decoction	Shr	Home garden	0.50

No.	Species (Auemporn Junsongduang Vourcher no.)	Family	Local name	Symptoms and Ailments treated (Part used)	Preparation	Life forms and status	Source of Medicinal plant	UV
118	*Piper nigrum* L. (AJ-77)	Piperaceae	Prik thai	Tonic (Se)	Eat with food	ExC	Home garden	0.25
119	*Piper retrofractum* Vahl (AJ-146)	Piperaceae	Dee pee	Tonic (Se)	Eat with food	ExC	Home garden	0.25
120	*Pithecellobium tenue* Craib (AJ-125)	Fabaceae	Pa ya chang san	Tonic (S), Anemia (S)	Decoction	T	Home garden	0.50
121	*Plumbago indica* L. (AJ-90)	Plumbaginaceae	Jad moon plerng dang	Tonic (Wp)	Decoction	Shr	Home garden	0.25
122	*Plumbago zeylanica* L. (AJ-91)	Plumbaginaceae	Jadmoon plerng kao	Tonic (Wp)	Decoction	Shr	Protected forest, Community forest	0.25
123	*Polyalthia cerasoides* (Roxb.) Bedd. (AJ-132)	Annonaceae	Sai dan/Ma hae ton	Tuberculosis (S,R)	Decoction	T	Protected forest	0.50
124	*Polyalthia debilis* Finet & Gagnep. (AJ-205)	Annonaceae	Kun krung	Dyspepsia (R)	Decoction	Shr	Protected forest	0.25
125	*Pterocarpus indicus* Willd. (AJ-126)	Fabaceae	Pa doo	Wound (B)	Grind apply to wound	T	Protected forest	0.25
126	*Pterospermum littorale* Craib (AJ-89)	Malvaceae	Mai ham aow	Intoxication (S) , Tonic (S), Abscess (S)	Decoction, Grind apply to pok	T	Protected forest	0.75
127	*Pueraria candollei* Wall. ex Benth. (AJ-165)	Lythraceae	Gaow	Abscess (L)	Grind apply to wound	WC	Home garden	0.25
128	*Rhinacanthus nasutus* (L.) Kurz (AJ-222)	Acanthaceae	Thong pan chang	Fever (R)	Decoction	Shr	Protected forest	0.25
129	*Rhodamnia dumetorum* (DC.) Merr. & L.M.Perry (AJ-207)	Myrtaceae	Kun tuey	Gastritis (R) , Fever (S), Hemorrhoids (S)	Decoction	ExShr	Home garden	0.75
130	*Ricinus communis* L. (AJ-78)	Euphorbiaceae	La hung	Postpartum women disorders (S,L)	Boil and Bath	Shr	Protected forest	0.25
131	*Rothmannia wittii* (Craib) Bremek. (AJ-147)	Rubiaceae	Mak mor	Lactation problem (S), Tonic (F,S,R), Muscle pain (R)	Decoction	Shr	Protected forest	1.75

No.	Species (Auemporn Junsongduang Vourcher no.)	Family	Local name	Symptoms and Ailments treated (Part used)	Preparation	Life forms and status	Source of Medicinal plant	UV
132	*Salacia chinensis* L. (AJ-79)	Celastraceae	Ta gai	Tonic (B), Diarrhea (S,R)	Decoction	Shr	Protected forest	0.75
133	*Schefflera leucantha* R.Vig. (AJ-153)	Araliaceae	Hanuman Prasanguy	Tuberculosis (L) , Asthmatic (L)	Decoction	Shr	Protected forest	0.5
134	*Scindapsus officinalis* (Roxb.) Schott (AJ-166)	Araceae	Ploo chang	Swelling (L)	Soaked and Bath	WC	Community forest	0.5
135	*Scleria levis* Retz. (AJ-88)	Cyperaceae	Ya kom pao	Gallstones (R)	Decoction	H	Community forest	0.25
136	*Scleropyrum pentandrum* (Dennst.) Mabb. (AJ-127)	Santalaceae	Nom wua /Nom sao	Lactation problem (S), Tonic (S,R),	Decoction	T	Community forest	0.75
137	*Schrebera swietenioides* Roxb. (AJ-87)	Oleaceae	Kok don	Head lice (S,R)	Grind with water and hair wash	T	Protected forest	0.25
138	*Sindora siamensis* Teijsm. ex Miq. (AJ-206)	Fabaceae	Tae	Gastritis (R)	Decoction	T	Community forest	0.25
139	*Smilax corbularia* Kunth (AJ-167)	Smilacaceae	Kao yen neua	Postpartum women disorders (S,Rh)	Decoction	WC	Community forest	0.25
140	*Smilax glabra* Roxb. (AJ-128)	Smilacaceae	Kao yen tai	Haemorrhage (R), Swelling (R)	Decoction	WC	Home garden	0.5
141	*Solanum trilobatum* L. (AJ-148)	Solanaceae	Ma kang kom	Cough (F)	Eat as fresh	WC	Community forest	0.25
142	*Stereospermum tetragonum* DC. (AJ-223)	Bignoniaceae	Care foid	Leucorrhea (S)	Decoction	T	Community forest	0.25
143	*Streblus asper* Lour. (AJ-152)	Moraceae	Koi	Body pain (Rh)	Mashed and apply	T	Protected forest	0.25
144	*Streptocaulon juventas* (Lour.) Merr. (AJ-168)	Apocynaceae	Pa song	Fever (Rh)	Decoction	WC	Community forest	0.25
145	*Strychnos nux-blanda* A.W. Hill (AJ-224)	Loganiaceae	Sang beua	Fever (S)	Decoction	T	Protected forest	0.25
146	*Strychnos nux-vomica* L. (AJ-80)	Loganiaceae	Tom ga	Fever (S)	Decoction	T	Community forest	0.25

No.	Species (Auemporn Junsongduang Vourcher no.)	Family	Local name	Symptoms and Ailments treated (Part used)	Preparation	Life forms and status	Source of Medicinal plant	UV
147	*Tetracera loureiri* (Finet. & Gagnep.) Pierre ex Craib (AJ-131)	Dilleniaceae	Lin had	Fever (L, Rh)	Decoction	WC	Home garden	0.25
148	*Thunbergia grandiflora* (Roxb. ex Rottl.) Roxb. (AJ-149)	Acanthaceae	Dok nam yae	Lactation problem (S,R)	Decoction	WC	Home garden, Community forest	0.25
149	*Thunbergia laurifolia* Lindl. (AJ-86)	Acanthaceae	Jang jeud	Intoxication (L,R), Diabetes (F, R,Sh)	Decoction	WC	Protected forest	0.75
150	*Tiliacora triandra* (Colebr.) Diels (AJ-*172)*	Menispermaceae	Ya nang kaew	Diabetes (L)	Decoction	WC	Home garden	0.25
151	*Tinospora crispa* (L.) Hook. f. & Thomson (AJ-150)	Menispermaceae	Bora pet	Tonic (S), Diabetes (S), Intoxication (S), Jaundice (S),	Decoction	WC	Community forest	1.5
152	*Trachelospermum asiaticum* (Siebold & Zucc.) Nakai (AJ-169)	Apocynaceae	Dua din	Diarrhea (R)	Decoction	WC	Community forest	0.25
153	*Trigonostemon reidioides* (Kurz) Craib (AJ-129)	Euphorbiaceae	Lod ta nong dang	Asthma (S) , Swelling (S)	Decoction,Grind with water and drink	Shr	Community forest	0.5
154	*Vietnamosasa pusilla* (A.Chev. & A.Camus) T.Q.Nguyen (AJ-170)	Poaceae	Pek	Gastritis (Wp) , Flatulence (Wp)	Decoction	Shr	Community forest	0.25
155	*Vitex glabrata* R.Br. (AJ-81)	Lamiaceae	Kai nao yai	Fever (R), Urination difficulty (S)	Ground with water and drunk	T	Protected forest	0.5
156	*Vitex pinnata* L. (AJ-151)	Lamiaceae	Ten nok	Gastritis (S, L), Fever (B,S)	Decoction	T	Protected forest	0.5
157	*Wrightia arborea* (Dennst.) Mabb. (AJ-158)	Apocynaceae	Mok yai	Cancer (F)	Decoction	Shr	Around village	0.25
158	*Wrightia religiosa* (Teijsm. & Binn.) Benth. ex Kurz (AJ-130)	Apocynaceae	Mok (mu-ja))	Cancer (S)	Decoction	Shr	Protected forest	0.25
159	*Xantonnea parvifolia* (Kuntze) Craib (AJ-82)	Rubiaceae	Giang peun	Fever (S), Jaundic (R), Tonic (W)	Grind with water and drink	T	Community forest	0.75

160	*Zingiber montanum* (J.Koenig) Link ex A.Dietr. (AJ-83)	Zingiberaceae	Prai	Flatulence (R),Tonic (S)	Decoction	H	Home garden	0.75
161	*Zingiber officinale* Roscoe (AJ-84)	Zingiberaceae	Kink	Flatulence (Rh)	Decoction	H	Home garden	0.5
162	*Ziziphus oenopolia* (L.) Mill. (AJ-85)	Rhamnaceae	Leb maew	Hemorrhoids (R), Muscle pain (S), Abscess (F)	Decoction	C	Community forest	0.75

Part used: B = Bark, F = Fruit, FL = Flower, R = Root, Rh = Rhizome, Sh = Shoot, S = Stem, Se = Seed, Wp = Whole plant
Life form: H = Herb, Shr = Shrub, T = Tree, C = Climber; *Native or exotic:* Ex = Exotic
